# The therapy-conditioned host: a state-transition framework for second primary cancer evolution

**DOI:** 10.3389/fonc.2026.1966473

**Published:** 2026-09-09

**Authors:** Rosiane Batista Mastelari, Tiago Veiras Collares

**Affiliations:** 1 University Hospital, Federal University of Pelotas - HUBrasil, Pelotas, Brazil; 2 Postgraduate Program in Biotechnology, Center for Technological Development, Federal University of Pelotas, Pelotas, Brazil

**Keywords:** cancer interception, cancer survivorship, clonal hematopoiesis, clonal selection, second primary cancer, somatic evolution, therapy-related mutagenesis, tissue ecology

## Abstract

Second primary cancers (SPCs) are an important challenge in cancer survivorship, yet their heterogeneous biological origins cannot be reduced to treatment exposure. Inherited susceptibility shared environmental and tissue-field effects, ageing, pre-existing somatic mosaicism and treatment-associated mutagenesis contribute to SPC risk. High-resolution studies show that therapy can alter somatic selection, clonal architecture and persistent immune or stromal states in normal tissues. We propose the therapy-conditioned host as a State-Transition framework integrating these observations in survivorship. In this framework, a pre-treatment host state (H_0_) is perturbed by therapy (P), generating evolving post-treatment states H(t), defined by interacting genomic, clonal, immune and stromal/tissue features. H(t) describes biological state at time t, whereas the sequence H_0_→H(t_1_)→H(t_2_)… defines the host trajectory. Equivalent exposures may generate different trajectories, while distinct treatments may converge on similar biological states. Treatment history is therefore an imperfect proxy for biological consequence. The framework does not imply that all SPCs are therapy-induced, that post-treatment conditioning is inherently carcinogenic, or that clonal expansion is equivalent to malignant progression. It proposes that the magnitude, direction and persistence of treatment-associated changes may influence the evolutionary opportunities of normal and premalignant cells. We outline testable predictions and a translational pathway requiring longitudinal validation, incremental predictive value beyond baseline susceptibility and treatment history, clinical actionability and evidence that intervention improves outcomes without disproportionate harm. The central question is not only what treatment a survivor received, but what biological state it produced, how that state evolves over time, and whether its trajectory meaningfully alters subsequent cancer risk.

## 1 The unfinished biology of cancer survivorship

Improved cancer survival has created a growing population in whom oncological risk does not end with control of the first malignancy. In a nationwide cohort of 457,334 survivors, 56,114 developed an SPC, with cumulative incidence rising from 6.3% at 5 years to 13.5% at 15 years (1). Yet SPC is a clinical category, not a single biological entity: a new malignancy can emerge through distinct histories of susceptibility, exposure and somatic evolution.

Risk models appropriately incorporate age, germline predisposition, environmental exposures, the first cancer and previous treatment. Recent survivor cohorts show substantial and interacting contributions from treatment and inherited susceptibility (2, 3); germline pathogenic variants also stratify second breast-cancer risk in young survivors (4). Genomic analyses further identify therapy-related, shared-developmental and independent origins of second malignancies (5). SPC should therefore be viewed as a convergent outcome rather than a uniform mechanism.

Treatment exposure itself has also acquired a richer biological meaning. High-depth sequencing of cancer-free tissues demonstrates treatment-associated mutagenesis and tissue-specific positive selection across normal organs (6), while single-cell studies show persistent mutational and clonal consequences in normal blood (7, 8). These findings do not establish causation for solid SPCs, but they demonstrate that normal tissues after therapy can differ biologically from their pre-treatment state.

**Figure 1:**
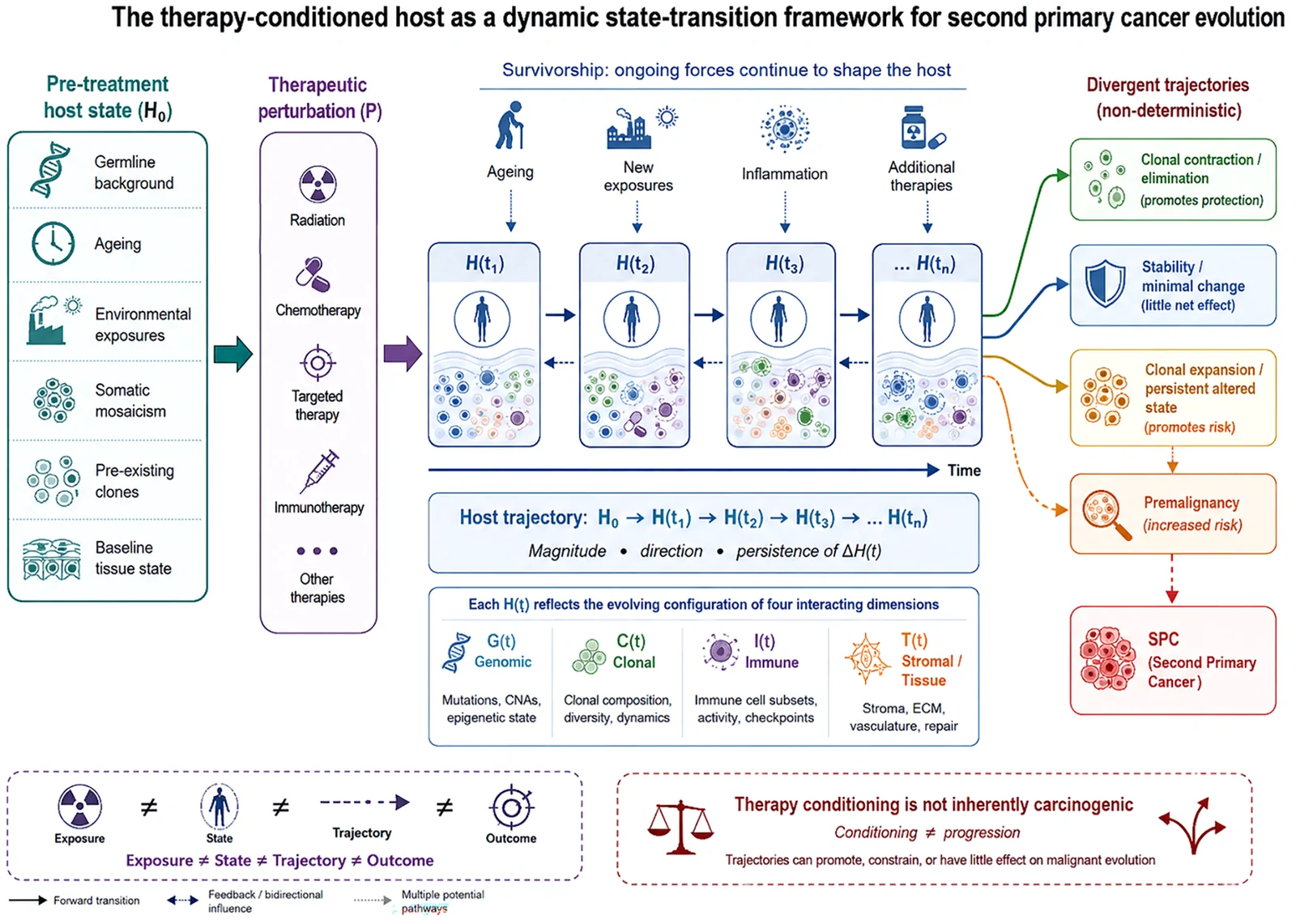
The therapy-conditioned host as a dynamic state-transition framework for second primary cancer evolution. A pre-treatment host state (H_0_), shaped by germline background, ageing, environmental exposure, somatic mosaicism, pre-existing clones and baseline tissue state, is perturbed by cancer therapy (P). Rather than defining a fixed post-treatment state, the framework represents serial host states H(t_1_), H(t_2_), … H(t_n_), each reflecting interacting genomic, clonal, immune and stromal/tissue dimensions. ΔH(t) denotes component-specific changes from baseline, not a scalar score. The sequence of states defines a trajectory that can show contraction or elimination, stability, expansion or persistent alteration, premalignancy, or SPC. The framework distinguishes therapeutic exposure, biological state, longitudinal trajectory and clinical outcome and does not imply that conditioning is inherently carcinogenic or that all SPCs are therapy-induced.

This Perspective asks whether SPC research should distinguish therapeutic exposure from the biological state produced by that exposure. We call this evolving state the therapy-conditioned host. Mutation, selection, field effects, tumor promotion, senescence and host responses are established concepts; the proposed contribution is not a new carcinogenic mechanism, but a shared longitudinal vocabulary that separates perturbation, measurable host state, trajectory and outcome. Its central hypothesis is that serial post-treatment biology may contain information not inferable from treatment history alone and should be considered for intervention only after clinical validation. The proposed state-transition framework is illustrated in **Figure 1**, and the supporting evidence map, conceptual boundaries, and translational guardrails are summarized in **Table 1**.

**Table 1:** Evidence map, conceptual boundaries and translational guardrails for the therapy-conditioned host framework.

Evidence domain	Representative evidence	Interpretation/boundary for SPCs	Key refs
Clinical burden & host susceptibility	Large survivor cohorts and germline/polygenic studies show heterogeneous SPC risk and treatment-host interaction.	Baseline susceptibility modifies, but does not define, the post-treatment state.	(1–5)
Normal-tissue somatic mosaicism	Driver-mutant clones and positive selection are common in normal epithelia and blood.	Therapy acts on a pre-existing evolutionary substrate; clonal expansion is not equivalent to cancer causation.	(11–15, 20, 22, 23, 28)
Therapy-associated mutagenesis	Treatment-specific mutational signatures occur in normal tissues and subsequent neoplasms.	Therapy can create variation available for future selection, but mutation burden alone does not predict SPC.	(5–8, 16–18)
Therapy-dependent selection & CH	Cytotoxic and immune therapies alter clone fitness; pre-existing DDR clones can expand, and selection can be pharmacologically modified.	Strongest lineage evidence is in hematopoiesis; generalization to solid SPCs remains unproven.	(6, 7, 20, 21, 24–28)
Persistent non-genetic conditioning	Stromal epigenetic memory, treatment-related epigenomic changes and senescence-mediated niche effects are measurable.	Immune and stromal states are plausible modifiers of premalignant fitness; direct causal links to solid SPCs remain limited.	(29–32)
Modern therapeutic paradigms	Immune checkpoint blockade, PARP inhibition and CAR T-cell therapy illustrate heterogeneous subsequent-neoplasm signals and mechanisms.	Association must be separated from causal pathway and from competing prior exposures.	(33–37)
Conceptual boundary	Field cancerization is spatial; tumor promotion is a selective mechanism; host-response literature largely addresses the existing tumor.	The therapy-conditioned host integrates relevant consequences around future independent carcinogenesis during survivorship.	(9, 10, 20, 21, 38)
Prospective state-transition hypothesis	Serial genomic, clonal, immune and tissue-state measurements before and after defined treatment.	Test whether ΔH(t) and H(t) add information beyond baseline susceptibility and treatment history; do not assume clinical utility.	Framework proposed here
Clinical translation boundary	Baseline CH is associated with subsequent malignancy in selected cohorts, but therapy-induced ΔH(t) and state-based SPC screening remain unvalidated.	Require analytical validity, prospective clinical validity, incremental predictive value and demonstrated actionability before surveillance.	(39–41)

## 2 Second primary cancers as convergent outcomes of distinct biological routes

SPCs can arise through several overlapping routes. Inherited susceptibility may independently predispose to multiple cancers and modify treatment effects; the interaction between polygenic susceptibility and radiotherapy in childhood cancer survivors provides population-level support for this principle (3).

Shared environmental or tissue-field exposures can also generate independent neoplastic lineages. Field cancerization describes spatial expansion of cancer-primed populations (9, 10), and driver-mutant clones are common in histologically normal oesophagus, bladder, stomach and bronchial epithelium (11–15). Such altered fields may predate both treatment and the first clinically detected cancer.

Pre-existing somatic mosaicism provides another route. In childhood second malignancies, some tumor pairs shared an early somatic event whereas others arose independently (5). Temporal sequence alone therefore cannot establish therapeutic causation.

Direct therapy-associated mutagenesis is supported by treatment-specific footprints in normal tissues and subsequent neoplasms (16–18). In 200 subsequent neoplasms from childhood cancer survivors, radiation-associated rearrangements and chemotherapy-related signatures remained detectable after a median latency of 26.4 years (18). Separately, whole-genome analysis of 611 childhood tumor genomes showed substantial therapy-associated mutagenesis in pretreated and relapsed tumors (19); because relapse represents evolution of the original malignancy, this finding supports treatment mutagenicity in surviving tumor cells rather than an SPC mechanism.

These routes are not mutually exclusive. Germline susceptibility, pre-existing clones, environmental fields and therapy-associated mutation or selection can coexist in one survivor. The therapy-conditioned host is therefore not a fifth cause of SPC, but a transversal framework for asking how treatment changes the biological substrate in which later independent carcinogenesis may occur.

## 3 Cancer therapy as a perturbation of somatic evolution

Therapy-associated carcinogenesis has classically been linked to DNA damage, and this mechanism remains central. Treatment-specific mutational footprints occur in tumors and histologically normal cells, including adult stem cells from healthy colon and liver exposed to common anticancer therapies (16, 17).

Recent work shows that therapy also alters selection. In 168 cancer-free samples spanning 16 organs, exogenous exposures shaped both mutation acquisition and positive selection in a strongly tissue-dependent manner (6). More than 25% of driver mutations in normal tissue exposed to systemic treatment were attributable to therapy in that cohort. Immunotherapy was not detectably mutagenic yet was associated with selection involving PPM1D and TP53, illustrating that treatment can sculpt somatic evolution without creating the selected mutation (6).

Mutation supply and cellular fitness are therefore separable. Treatment can generate variants, eliminate competitors and change regenerative or immune pressures. Population-scale analyses show that positive selection in normal tissue is not synonymous with cancer causation (20), while evolutionary models of tumor promotion emphasize selection of initiated clones without obligatory mutagenesis (21). Our question is not whether therapy-dependent selection exists, but what it means for an independent cancer emerging years later.

Tissue specificity further cautions against using exposure as a uniform proxy for biological consequence. Even related platinum agents produce markedly different mutational burdens in normal blood cells (7). Drug, dose, tissue, timing, host genotype and regenerative context must therefore be considered when defining the post-treatment state.

## 4 Pre-existing clones and therapy-dependent fitness: lessons from hematopoiesis

Hematopoiesis provides the strongest human proof of principle that therapy can alter the fitness of pre-existing normal-cell clones. Age-related clonal hematopoiesis predicts hematological malignancy (22, 23), cytotoxic therapy enriches DNA-damage-response clones (24), and long-term pediatric cancer survivors can retain therapy-related CH decades after exposure (25).

Single-cell studies add temporal resolution. Mitchell and colleagues showed durable chemotherapy-associated mutation burdens and altered blood-cell population structure; phylogenetic reconstruction indicated that some expanded PPM1D-mutant clones pre-dated therapy, supporting selective amplification rather than obligatory *de novo* creation (7).

Uryu and colleagues sequenced 1,276 single-cell-derived hematopoietic stem and progenitor colonies after chemotherapy and autologous transplantation. Treatment reduced clonal diversity, generated parallel high-risk clones and, in matched therapy-related myeloid neoplasms, linked the malignant lineage to a TP53-mutant HSPC clone (8).

This selective process is potentially modifiable. In randomized trials, transient CDK4/6 inhibition with trilaciclib reduced chemotherapy-associated expansion of DNA-damage-response CH; most post-treatment mutations were already detectable before therapy at low abundance (26). This is proof of principle that therapy-driven clonal fitness can be altered, not proof that SPC can be prevented.

Host genotype further modifies clonal fitness (27), and somatic phylogenies show that normal blood remains dynamically clonal throughout life (28). The selective value of a mutation therefore emerges from interactions among somatic genotype, germline background and environmental pressure.

CH should nevertheless remain a proof of principle rather than a universal template. Blood permits serial, high-sensitivity sampling and lineage reconstruction that are rarely feasible in solid tissues. Comparable evidence connecting therapy-conditioned normal clones to later solid SPCs is still lacking.

A central question is which principles demonstrated in hematopoiesis generalize to solid tissues. Rather than assuming an identical model, future studies should test therapy-dependent selection, longitudinal clonal remodeling and analogous persistent tissue-state changes in relation to subsequent solid-SPC trajectories.

## 5 Persistent non-genetic conditioning of the post-treatment host

Mutation and clonal selection do not capture the full biological consequence of therapy. Stromal, immune and regenerative compartments can retain functional changes after treatment, although direct evidence that these states cause solid SPCs remains limited.

Human survivor data demonstrate persistent non-genetic memory. Dermal fibroblasts exposed to radiotherapy retain epigenetic alterations associated with impaired repair (29), and long-term DNA-methylation changes have been documented after childhood cancer therapy (30). These findings establish persistence, not carcinogenic causation.

Experimental models provide a mechanistic bridge to clonal fitness. Therapy-induced senescence can impair antitumor immune surveillance (31), while senescent bone-marrow stromal cells increase the fitness of DNMT3A-mutant hematopoietic clones; senescent-cell depletion reduced clonal burden and delayed myeloid progression (32). Direct relevance to therapy-conditioned solid-tissue SPCs remains to be shown.

Immune conditioning illustrates why post-treatment change should not be assigned a fixed direction. A retrospective patient-level cohort associated checkpoint therapy with lower SPC incidence (33), but non-randomized allocation and limited latency preclude a preventive interpretation. Conversely, a 2026 SEER analysis of 185,755 patients across four metastatic cancers found no significant change in SPC incidence between pre- and post-ICI eligibility eras; the study used treatment era rather than individual ICI exposure and had shorter follow-up in post-ICI groups, and its authors cautioned that earlier associations may reflect shared risk factors rather than treatment-induced immunomodulation (34). Together with evidence that immunotherapy can alter normal-tissue selection without detectable mutagenesis (6), these discordant observations support mechanism-specific study rather than assuming that immune conditioning is carcinogenic or protective.

Modern therapies further illustrate the need for mechanism-specific attribution. PARP inhibitors have been associated with therapy-related myeloid neoplasms (35), whereas second T-cell malignancies after CAR T-cell therapy are rare and mechanistically heterogeneous, potentially involving prior therapy, clonal hematopoiesis, viral biology or vector integration (36, 37).

We therefore use tissue permissiveness only as a candidate property of H(t): the degree to which immune, stromal and regenerative conditions permit clonal persistence or expansion. Earlier host-response literature established that cancer therapy can induce systemic and local responses that support an existing tumor (38). The therapy-conditioned host differs in unit of analysis and endpoint: it focuses on persistent biology in survivors and asks whether post-treatment normal-tissue states relate to future independent carcinogenesis. Persistent tissue memory and therapy-associated selection are documented; causal linkage of these states to most solid SPCs is not.

## 6 The therapy-conditioned host as a state-transition framework

We propose the therapy-conditioned host as a survivorship-focused unit of analysis, not a new molecular mechanism or mathematical model. H_0_ denotes the pre-treatment host state, P the therapeutic perturbation and H(t) the biological state at time t after treatment, described across genomic G(t), clonal C(t), immune I(t) and stromal/tissue T(t) dimensions. The notation provides a shared vocabulary that explicitly separates exposure from state and outcome; it does not imply that these dimensions are additive, commensurable or reducible to a validated score.

Exposure and state need not map one-to-one: similar regimens may yield distinct H(t) configurations, whereas different perturbations may converge on similar states. Thus, treatment history identifies P, whereas H(t) describes an individualized biological consequence. Therapy conditioning is directionally agnostic: treatment-associated states may promote, constrain or have little effect on subsequent malignant evolution. SPC remains one possible outcome, not an intrinsic property of H(t).

The framework is deliberately agnostic about individual causality. An SPC may arise predominantly through inherited susceptibility, shared exposure or independent stochastic evolution. The testable proposition is narrower: treatment-associated changes in host biology may alter subsequent evolutionary trajectories. Selection is not causation, state is not outcome and biological detectability is not clinical actionability.

## 7 From state transition to evolutionary trajectory

A state-transition framework requires serial biology: H_0_ before treatment, P during therapy and H(t) across survivorship. Here ΔH(t) is shorthand for the set of component-specific changes from baseline—not a scalar, computable index. Any future composite score would require independent derivation and validation. H(t) denotes state at one time point; H_0_→H(t_1_)→H(t_2_)→… denotes trajectory. Serial measurements can therefore distinguish transient from persistent or progressively evolving changes.

Time is essential because components evolve at different rates: mutational scars may become fixed after exposure, whereas clone size, immune constraints and stromal states can change for years. Longitudinal sampling is needed to establish temporal precedence and to separate durable trajectories from transient treatment responses.

The framework also predicts path dependence: the meaning of a measured H(t) may depend on how that state was reached. Similar baseline states and treatments may diverge through pre-existing clone abundance, tissue exposure, regeneration, inflammation or later environmental history, while different therapies may converge on similar states. The added value of the framework therefore lies in testing trajectory-level biology that treatment history alone cannot encode.

## 8 From treatment history to measurable state: a responsible translational path

Current SPC risk assessment is dominated by historical and clinical variables. Baseline host state already has prognostic relevance, but evidence for therapy-induced change in state is absent. In 63,690 individuals with a first primary cancer, pre-diagnostic clonal hematopoiesis was associated with a modestly higher second-cancer risk (HR 1.3, 95% CI 1.2-1.5) during a median 3.9 years of follow-up (39). Because CH preceded both the first cancer and treatment, this study concerns H_0_, not ΔH(t); the short follow-up also limits inference for long-latency solid SPCs and reinforces the need for careful endpoint adjudication.

Similarly, in 1,931 autologous HCT recipients, CH measured in pre-HCT mobilized blood products was associated with nonmyeloid subsequent malignant neoplasms (HR 1.72, 95% CI 1.15-2.59), with risk increasing with clone size (40). This too measures a baseline state before conditioning therapy. The direct empirical gap is therefore explicit: baseline clonal state can carry prognostic information, whereas no study cited here has shown that therapy-induced ΔH(t) provides incremental causal or predictive information beyond H_0_ and treatment history.

Candidate measurements include clonal hematopoiesis or tissue-specific driver clones, treatment-associated mutational signatures, cfDNA, immune profiling, epigenetic treatment memory and stromal or inflammatory markers. Informative cohorts require defined exposures, preferably pre-treatment biospecimens, serial post-treatment sampling, adjudicated SPC endpoints and control for recurrence, competing mortality, germline predisposition and environmental risk. No single biomarker should be assumed to represent H(t).

The translational target need not be a static H(t) biomarker. Clonal growth rate, persistence of epigenetic states or sustained immune/stromal remodeling may be more informative than single-time-point values, but this is a hypothesis to test rather than an established predictive advantage. Current CH guidance likewise emphasizes risk-based interpretation rather than indiscriminate screening (41).

A direct longitudinal test would compare H_0_ with serial H(t_1_)…H(t_n_) across survivors following distinct trajectories. It should establish whether component-specific changes emerge after defined therapy, persist or evolve, precede premalignant or SPC endpoints and add information beyond baseline susceptibility and exposure. Where feasible, molecular or lineage continuity could test evolutionary linkage; predominantly transient changes or lack of incremental information would limit the framework.

Such analyses require methods matched to repeated measurements and delayed outcomes. Landmark approaches or joint longitudinal-time-to-event models can update risk as new H(t) measurements accrue (42, 43), while competing-risk methods should account for death that precludes SPC (44). Risk sets must begin only after the relevant state measurement is available to avoid immortal time bias (45), and surveillance intensity should be measured or standardized because differential follow-up can inflate detected SPC incidence (46). Recurrence, metastasis and subsequent treatment should be adjudicated and, where appropriate, modeled as time-varying or intercurrent events.

Clinical translation should still require four thresholds before state-based surveillance is adopted: analytical validity; prospective clinical validity for a defined SPC endpoint; incremental value beyond established risk factors; and actionability supported by evidence that altered surveillance or intervention improves meaningful outcomes. Detection alone can create anxiety, incidental findings, unnecessary procedures and overmedicalization without proven benefit.

## 9 Testable predictions, research priorities and translation guardrails

Prediction 1 — Exposure-state discordance. Among survivors matched by tissue and therapeutic exposure, serial component-specific changes should show reproducible between-survivor divergence exceeding prespecified technical and repeat-measure variability. Conversely, distinct exposures may converge on similar states. If observed changes are indistinguishable from measurement variability or fully explained by exposure and tissue alone, exposure-state discordance would not be supported.

Prediction 2 — Baseline-state dependence. Pre-treatment genomic, clonal, immune or tissue features should explain a prespecified incremental proportion of variation in subsequent component trajectories, or show reproducible treatment-by-H_0_ interactions, beyond exposure alone (3, 7, 26, 27). Failure to improve model fit or prediction in independent validation would argue against baseline-state dependence.

Prediction 3 — Trajectory-level information. Longitudinal H(t) features should improve prediction of subsequent premalignant or SPC outcomes beyond H_0_ and treatment history by a prespecified minimum increment in discrimination, calibration, prediction error or clinical net benefit. If the confidence interval excludes that minimum gain in prospective external validation, the proposed predictive value of trajectory would be unsupported.

Prediction 4 — Tissue- and therapy-specific trajectories. Prespecified therapy-tissue strata should show reproducible differences in component trajectories after adjustment for baseline state and measurement variability (6, 7, 33–37). If standardized trajectories are indistinguishable across relevant strata, tissue- or therapy-specific conditioning would not be supported for those comparisons.

Prediction 5 — State modifiability. In controlled perturbation studies, an intervention should change a prespecified H(t) component by a prespecified biologically meaningful amount relative to control (26, 32). Failure to meet that effect would refute modifiability for that component and intervention. Any reduction in SPC risk remains a separate endpoint and must not be inferred from biomarker change.

The research priority is staged translation: establish longitudinal survivor cohorts; resolve tissue- and therapy-specific effects; integrate germline susceptibility with somatic and ecological state; test temporal and, where possible, lineage continuity; identify reproducible high-risk trajectories; and only then test intervention. Equity must be considered throughout, because costly or poorly interpretable molecular surveillance could widen disparities in survivorship care.

## 10 Discussion — what treatment leaves behind, and what we still must prove

Cancer therapy can leave mutational footprints in normal tissues, alter somatic selection and clonal architecture, and produce persistent non-genetic changes (6–8, 29–32). Baseline clonal states can also be associated with later second malignancies (39, 40). What remains unproven is the central construct proposed here: that therapy-induced change in an integrated post-treatment host state carries incremental information about SPC evolution.

The therapy-conditioned host is therefore an organizing framework and shared vocabulary, not a validated score. Treatment history describes P; H(t) describes biological state; ΔH(t) denotes component-specific change; and H_0_→H(t_1_)→H(t_2_)→… defines a trajectory whose relationship to malignant evolution must be demonstrated. Its value depends on whether these distinctions generate measurements and predictions that outperform simpler exposure-based descriptions.

The clinical ambition remains conditional and patient centred. State-based surveillance would be justified only if it improves risk stratification and leads to monitoring or intervention with greater benefit than harm. Until that evidentiary chain is achieved, the framework’s value is to define the empirical gap, the negative results that would weaken it, and the boundary between biological insight and clinical action.

## Data Availability

The original contributions presented in the study are included in the article/supplementary material, further inquiries can be directed to the corresponding author/s.
